# Metastatic rhabdomyosarcoma of the thyroid gland, a case report

**DOI:** 10.1186/1758-3284-4-27

**Published:** 2012-05-29

**Authors:** Mohamed T Hafez, Mohamed A Hegazy, Khaled Abd Elwahab, Mohammad Arafa, Islam Abdou, Basel Refky

**Affiliations:** 1 Surgical oncology unit, Oncology center, Mansoura University, Mansoura, Egypt; 2 Pathology Department, Mansoura Faculty of Medicine, Surgical oncology unit, Oncology center, Mansoura University, Mansoura, Egypt

## Abstract

The thyroid gland is a known but an unusual site for metastatic tumors from various primary sites. Despite the fact that it is one of the largest vascular organs in the body, clinical and surgical cases have given an incidence of 3 % of secondary malignances of the organ. Nevertheless, thyroid metastases are not an exceptional finding at autopsy, they are encountered in 2 % to 24 % of the patients with malignant neoplasm.

Soft tissue sarcomas metastatic to the thyroid are extremely rare as the majority of thyroid metastasis are caused by tumors of the kidneys, lungs, mammary glands, ovaries , and colon or by melanomas.

We report a case of 22-years-old woman with right leg rhabdomyosarcoma metastatic to the thyroid gland.

## Case report

### Clinical history

A 22 years old pregnant female (14 weeks) presented with a rapidly growing soft tissue mass in the right leg. Pre operative MRI showed a 7x6x5 cm soft tissue mass at the anterior compartment of the right leg indenting the neurovascular bundle.

The patient was willing to keep her precious pregnancy. Chest X-Ray with lead shield was performed and was free.

Patient underwent anterior compartmental resection of the right leg with the post operative pathology proved to be high grade pleomorphic sarcoma consistent with rhabdomyosarcoma.

The patient was referred to the medical oncology unit where she agreed to do abortion (16 week) to start … chemotherapy.

During her second follow up visit- one month later- at our surgical oncology outpatient clinic , she complained of dyspneaic manifestations with neck swelling.

Neck ultra sound revealed an enlarged right thyroid lobe with hypoechoic heterogenous nodule measuring 4 x 2 cm with other 2 small hypoechoic nodules in the same lobe. FNAC from the thyroid nodule proved to be undifferentiated malignant tumor. All biopsies were sent to the pathology laboratory, processed and reported as routine paraffin embedded tissues. Routine histopathological examination was performed on thin sections (5 μm) stained with H&E.

**Figure 1:**
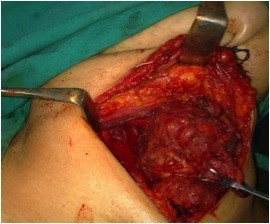
Total Thyroidectomy, the right lobe shown to be large with infiltration of Rt common carotid artery.

Patient underwent total thyroidectomy, which showed to be difficult due to infiltration of the right common carotid artery. Figure 1

## Postoperative pathology

### Pathological findings

#### Gross appearance

Cut surface from the tumor in the right leg was formed of tissue mass covered by ellipse of skin. On dissection, it was firm mass measuring about 7x6x5 cm with heterogenous grayish white to tan brown cut surface and surrounded by muscle fibers.

The thyroidectomy specimen showed the right lobe to be enlarged compared to left lobe, having irregular surface and, on cut section, showed firm grayish white infiltrative growth.

#### Light microscopy

The tumor of the right leg appeared as high-grade malignant tumor tissue. It was formed of intersecting fasicles and diffuse proliferation of highly atypical spindle shaped to polygonal tumor cells. They exhibited moderate amount of eosinophilic cytoplasm, vesicular nuclei and prominent nucleoli. Some tumor giant cells were detected. Large areas of necrosis and high mitotic activity were present.

Immunohistochemical staining was performed using antibodies against vimentin, desmin, s100, SMA and CK. The procedures were done according to the maunfacture’s instructions (Dako,Glostrup, Denmark). The tumour cells’ cytoplasm were positively stained for vimentin, desmin and SMA. No immunoreactivity was observed for CK or S100. Vimentin immunoreactivity was found to be more diffusing compared to that of desmin.

The subsequent thyroid FNA revealed, in cell block preparations, malignant cells of similar nature as described in the original specimen. The cells were positive for vimentin and negative for CK, a picture confirming the sarcomatous nature of the neoplasm.

**Figure 2:**
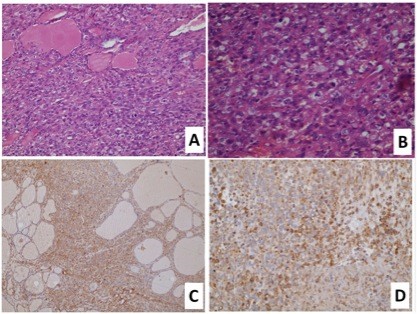
The sarcoma tissue invades between the thyroid follicles (H&E x100) (A). The tumour cells show significant pleomorphism (H&E x200), (B), diffuse immunoreactivity to vimentin (DAB peroxidase x100) (C) and desmin (DAB peroxidase x200) (D).

The thyroidectomy specimen showed diffuse infiltration of the thyroid tissue by similar tumour (Figure 2A &2B), which showed also identical immunorecativty to the original specimen. There were positivity for vimentin, desmin (Figure 2C &2D) and negative CK staining.

All the procedures of this work were done following the approval of the ethical committee of Mansoura University.

## Discussion

Rhabdomyosarcoma is a malignant soft-tissue sarcoma believed to develop from undifferentiated mesenchymal cells destined for a skeletal muscle lineage [1,2]. It is a highly aggressive tumor with a tendency for advanced and disseminated disease early in its course [3]. Whereas rhabdomyosarcoma is a common childhood malignancy, it is exceedingly rare in adults, accounting for around 2 % to 5 % of adult soft-tissue sarcomas [4].

Though uncommon in adults, rhabdomyosarcoma tends to be more aggressive and more resistant to chemotherapy than its childhood counterpart [4].

Metastasis to the thyroid gland is not as rare as previously believed. Its incidence has been shown, in autopsy series, to be more than the incidence of primary thyroid malignancy [5].

The overall incidence, not surprisingly, varies from 1.25 % in unselected autopsy series to 24 % in autopsy of patients with widespread malignant neoplasm [6].

In both clinical and autopsy series, renal cell, breast and lung carcinomas are the most frequent sources of metastases to the thyroid [7,8]. Although thyroid metastases are possibly more common than primary thyroid carcinoma (as indicated by autopsy series), they are less of a clinical problem [9].

Thyroid metastasis may be the initial evidence of disease or perhaps the first presentation of recurrent disease [9].

Thyroid nodules in a patient with a history of malignancy can pose a diagnostic challenge, particularly if they present many years after the initial tumour.. It has been the experience of other institutions that in a patient with a history of cancer, a malignant thyroid nodule is much more likely to be metastatic than a new primary tumour [10].

There is nothing clinically to differentiate thyroid metastases from primary thyroid cancer [10].

FNA biopsy could confirm a clinical suspicion of metastasis to the thyroid gland accurately with low morbidity; it also might help to avoid unnecessary thyroidectomy in patients with a poor prognosis [11]. In most instances there is abundant cellularity and the cells may be typical of the original site, especially when specific immunohistochemical stains are performed. Negative staining with antithyroglobulin and anti-calcitonin antibodies would favour a metastatic tumour [9].

Management depends on the primary site of the original tumour, presence of other metastases and symptoms caused by the thyroid mass [9]. Adequate surgical treatment may prove to be life prolonging or life saving [12].

Nakhjavani et al stated Shorter mean survival in patients who were treated non-surgically (25 months), compared to patients who underwent thyroidectomy alone or thyroidectomy with adjuvant therapy (34 months) was reported in one series [13].

## Conclusion

Any new thyroid nodule occurring in patients with a known malignancy, should be considered as a possible metastatisis until proved otherwise. FNAC is a good tool to diagnose a metastatic disease.
